# Holmium laser ablation of the prostate (HoLAP) with moses technology for the surgical treatment of benign prostatic hyperplasia

**DOI:** 10.1590/S1677-5538.IBJU.2021.0455

**Published:** 2021-09-10

**Authors:** Antonio Andrea Grosso, Fabrizio Di Maida, Andrea Mari, Samuele Nardoni, Agostino Tuccio, Andrea Minervini

**Affiliations:** 1 University of Florence Department of Experimental and Clinical Medicine Unit of Oncologic Minimally-Invasive Urology and Andrology Florence Italy Department of Experimental and Clinical Medicine, University of Florence, Unit of Oncologic Minimally-Invasive Urology and Andrology, Careggi Hospital, Florence, Italy

## Abstract

**Purpose:**

The expansion of technology is leading to a paradigm shift in several urological fields (1, 2). In particular, the adoption of lasers within the surgical treatment of patients with benign prostatic hyperplasia (BPH) is considered one of the most relevant innovations (3-5). In this video, we aimed to report our experience with holmium laser for the ablation of the prostate (HoLAP) in patients with obstructive lower urinary tract symptoms (LUTS) due to BPH.

**Materials and Methods:**

From 2018 to 2020, 10 patients with obstructive LUTS secondary to BPH were treated at our Institution with HoLAP (120W Holmium laser Lumenis^®^ with Moses^®^ technology). Main inclusion criteria were: 1) International Prostate Symptom Score ≥12; 2) prostate volume ≤65mL, 3) maximal flow rate (Qmax) ≤15ml/s at preoperative non-invasive uroflowmetry.

**Results:**

Mean patient age was 65 (range: 59-72) years. Preoperative mean prostate volume was 50 (range: 35-65) mL. Mean operative time was 66 (range: 45-85) minutes with a mean laser time/operative time ratio of 0.51 (range: 0.44-0.60). Voiding symptoms, Qmax and post voiding residual were significantly improved after 3 and 12 months (all p <0.05). No postoperative urinary incontinence was detected.

**Conclusions:**

The present findings suggest that HoLAP is a slightly time-spending procedure, thus its use should be limited to prostate volume <70-80mL. However, no postoperative complications were recorded at all. This technique showed to be a safe option in patients with low-intermediate prostate volume, also in patients whose antiaggregant/anticoagulant therapy is maintained.

## INFORMED CONSENT

Informed consent was obtained from all individual participants included in the study. All the procedures were in accordance with the ethical standards of the institutional and national research Committee and with the 1964 Helsinki declaration and its later amendments or comparable ethical standards.
